# Effect of climate change on sporulation of the teleomorphs of *Leptosphaeria* species causing stem canker of brassicas

**DOI:** 10.1007/s10453-015-9404-4

**Published:** 2015-09-14

**Authors:** Joanna Kaczmarek, Andrzej Kedziora, Andrzej Brachaczek, Akinwunmi O. Latunde-Dada, Sylwia Dakowska, Grzegorz Karg, Małgorzata Jedryczka

**Affiliations:** Institute of Plant Genetics, Polish Academy of Sciences, Strzeszynska 34, 60-479 Poznan, Poland; Institute of Agricultural and Forest Environment, Polish Academy of Sciences, Bukowska 19, 60-809 Poznan, Poland; DuPont Poland Ltd., Postepu 17b, 02-676 Warsaw, Poland; Rothamsted Research, Harpenden, Hertfordshire AL5 2JQ UK

**Keywords:** Aerobiology, Climate change, Blackleg, Spore concentration, Stem canker, Oilseed rape

## Abstract

*Leptosphaeria maculans and L. biglobosa* are closely related sibling fungal pathogens that cause phoma leaf spotting, stem canker (blackleg) and stem necrosis of oilseed rape (*Brassica napus*). The disease is distributed worldwide, and it is one of the main causes of considerable decrease in seed yield and quality. Information about the time of ascospore release at a particular location provides important data for decision making in plant protection, thereby enabling fungicides to be used only when necessary and at optimal times and doses. Although the pathogens have been studied very extensively, the effect of climate change on the frequencies and distributions of their aerially dispersed primary inoculum has not been reported to date. We have collected a large dataset of spore counts from Poznan, located in central-west part of Poland, and studied the relationships between climate and the daily concentrations of airborne propagules over a period of 17 years (1998–2014). The average air temperature and precipitation for the time of development of pseudothecia and ascospore release (July–November), increased during the years under study at the rates of 0.1 °C and 6.3 mm per year. The day of the year (DOY) for the first detection of spores, as well as the date with maximum of spores, shifted from 270 to 248 DOY, and from 315 to 265 DOY, respectively. The acceleration of the former parameter by 22 days and the latter by 50 days has great influence on the severity of stem canker of oilseed rape.

## Introduction

The frequency of accelerated changes in global climate has become accentuated during the last few decades the world over, and their effects have been observed in Poland as well (Millennium Ecosystem Assessment 2005; Kedziora et al. 2011; Kedziora and Kundzewicz 2011; Pachauri et al. 2014). These changes are manifest in increased temperature, alterations in the pattern and distribution of annual rainfall and in increased incidence of climate extremes (Cutter et al. 2012). Models have predicted variations and changes in temperature and precipitation distributions in different regions of the world. Temperature has been forecast to rise across the globe, particularly in the more northerly latitudes, but less so in the tropics (Pachauri et al. 2014). Precipitation will increase in regions, where it is currently high, but will decrease where currently low. Accelerated increase in temperature in Wielkopolska (Great Poland) that has been observed since 1980 has been accompanied by comparatively minor changes in precipitation. During the 60 years spanning 1950 and 2010, air temperature increased by 1.8 °C which translates into a temperature increase of about 0.3 °C per decade over this period. While this observation is much higher than was indicated by the Intergovernmental Panel on Climate Change (IPCC) reports, changes in precipitation during this time period amounted to only 20 mm; i.e. a 3-mm change per decade.

Long-term studies of patterns of air temperature and phenology of plants clearly show climatic effects on many physiological processes, such as an increased acceleration of time to flowering (Puc and Kasprzyk 2013; Bock et al. 2014), fruiting (Kasprzyk et al. 2014) or a growing risk of plant damage by late frosts in spring (Augspurger 2013). Climate change might cause both the appearance and the establishment of alien plant species and an increase in the populations of native species which might become harmful to other valuable species and to agricultural production (Bertelsmeier et al. 2013; West et al. 2012). Changes in climate do not only have an impact on plants and animals, but they also affect numerous microbial populations. The latter influence is often overlooked due, perhaps, to the comparatively small size of microorganisms, but the numerous, varied, important and critical biological functions of these life forms cannot be overemphasized. Bacteria, fungi and other microbes are indispensable as members and decomposers within all ecosystems worldwide. Numerous studies (Frey et al. 1999; Evershed et al. 2006; Rinnan et al. 2007; Gray et al. 2011; Dieleman et al. 2012; Guenet et al. 2012) have demonstrated that bacteria and fungi respond differently to multi-factorial climate change in a number of regions (Andresen et al. 2014). There is a need for undertaking further intensive studies to enable a clearer understanding of the impact of global climate change on microorganisms, including the populations of those that are pathogenic to plants. Moreover, the possibility of the acquisition of increased virulence and invasiveness, arising from climate change, by currently innocuous and avirulent species of bacteria and fungi, including insects and weeds as well, is a very serious threat to agricultural production (Hellmann et al. 2008).

Phoma stem canker (blackleg) is a damaging disease of oilseed rape (*Brassica napus* L.) worldwide (Fitt et al. 2006). It is caused by the dothideomycete fungi *Leptosphaeria maculans* (Desm.) Ces. et de Not. and *L. biglobosa* (Shoemaker and Brun 2001; anamorphs = *Plenodomus lingam* and *P. biglobosus* respectively). Both of these fungi have a pan-European distribution and mixed populations of *L. maculans* and *L. biglobosa* occur in western European countries including France (Penaud et al. 1999), Germany (Kuswinanti et al. 1999), the UK (Humpherson-Jones 1983) and Poland (Kaczmarek and Jedryczka 2011), although regional variations in the frequencies of the two species have also been reported (Jedryczka 2006; Stonard et al. 2010a, 2010b). The pathogens are often found together, on the same plants of oilseed rape (Williams and Fitt 1999), so they are commonly termed the *L. maculans*–*L. biglobosa* species complex (Mendes-Pereira et al. 2003). Both *L. maculans* and *L. biglobosa* have a similar life cycle (West et al. 2001). In Australia, Canada and Europe, epidemics of phoma stem canker are initiated by airborne ascospores released from pseudothecia that develop on stubble from the previous season’s crop of members of the family Brassicaceae (Hall 1992). These ascospores initiate epidemics in autumn and the speed of pseudothecial maturation and spore release depends on weather conditions to a great extent (Dawidziuk et al. 2012a). Pseudothecial development is accelerated at temperatures between 5 and 20 °C (Toscano-Underwood et al. 2003; Aubertot et al. 2006; Kaczmarek and Jedryczka 2008), and ascospores are released within the same temperature range (Huang et al. 2005). Ascospore release is also influenced by surface wetness and small concentrations of ascospores in the air can be observed after dews (Huang et al. 2005). However, ascospore showers are triggered by rainfall (Salam et al. 2003; Huang et al. 2005). Conducive summer and autumn conditions favour the development of severe stem canker epidemics (McGee 1977; Hall 1992). Splash-dispersed pycnidiospores (asexually produced conidia of the *Plenodomus* (=*Phoma*) anamorphs of these *Leptosphaeria* spp.) can also initiate disease epidemics in rapeseed crops (Barbetti 1976; Guo and Fernando 2005). However, they are largely regarded as secondary inocula (Travadon et al. 2007) in Europe and were not considered for enumeration in the current study.

Although both pathogens have been studied very extensively, the effect of climate change on the aerobiology of these causes of phoma stem diseases of rapeseed has not been addressed to date. The aim of the work undertaken in this report was to investigate seasonal fluctuations in the abundance and proportions of airborne inocula of the *L. maculans*–*L. biglobosa* species complex sampled over 17 consecutive OSR growing seasons in central Poland in relation to climate change. The effect of climate change on the sporulation of perfect stages (teleomorphs) of members of the *L. maculans*–*L. biglobosa* species complex was analysed over a long-term (1950–2014) meteorological span and over a comparatively shorter (1998–2014) period during which biological data in the form of airborne ascospores were collected, compiled and analysed for this study.

## Materials and methods

### Location of experimental site

Air samples were collected with volumetric air samplers (described below) in Poland from experimental plots at the Institute of Plant Genetics, PAS in Poznan (Greater Poland region, N52°24′28.0 E16°54′45).The sampling area was a suburb of the city, surrounded by a mixture of amenity grassland and medium-sized agricultural fields.

### Operation of spore samplers to collect ascospores of *L. maculans*–*L. biglobosa* species complex

Spores were collected daily in autumn from 1 September to 30 November in each of the years 1998–2014, using 7-day recording Hirst-type volumetric spore samplers (Hirst 1952; Burkard Manufacturing Co., Rickmansworth, UK) which were placed at ground level and surrounded by the stem debris of winter oilseed rape from the previous season’s crop that had been affected by phoma stem canker. The amount of debris around each sampler was ca. 0.35 m^−3^. The spore samplers were fitted with a 2 × 14 mm orifice that sampled 10 L of air min^−1^, so that any particles in the air were deposited onto a Vaseline-coated Melinex tape mounted on a rotating drum (1 revolution per week, turning at 2 mm h^−1^). The tape was changed every 7 days, and each tape was then cut into 48-mm-long (each representing 24 h) pieces. Each piece was further cut in half length-wise. One half (7 mm width) was mounted onto a microscope slide, stained with 0.1 % (w/v) trypan blue and examined with a light microscope. The numbers of spores was counted, and the daily ascospore concentration m^−3^ of air was estimated according to the formula produced by Lacey and West (2006).

### Climate of the region of study

The climate of the Wielkopolska region, Poland, is greatly influenced by the air masses from the north Atlantic, Eastern Europe and Asia (arctic 6 %, polar maritime 59 %, polar continental 28 % and tropical 7 %), which are further modified by strong Arctic and Mediterranean influences. These wind combinations result in highly changeable weather conditions. The predominance of westerly winds ushers in a strong oceanic influence which is manifested in milder winters and cooler summers than in the centre and east of Poland. In this region, the mean annual temperature is about 8 °C (ranging from 6.9 to 8.5 °C). Mean annual global solar radiation amounts to 3700 MJ m^−2^ and the mean annual net radiation is about 1315 MJ m^−2^(Kedziora and Kayzer 2012). Thus, thermal conditions existing in the Wielkopolska landscape are favourable for crop cultivation, with temperatures above 5 °C lasting over the growing season from 21 March till 31 October annually. The mean annual precipitation is 594 mm, of which 365-mm falls between April and September, and the remaining in the period from October to March. Decreasing the ratio of summer precipitation to winter precipitation will prove unfavourable for successful crop cultivation (Kedziora 2010).

### Meteorological data and statistical analyses

Meteorological data covering the entire study period were provided by the Institute for Agricultural and Forest Environment PAS, Poznan, Poland. Observations were carried out at the weather station located within the Turew landscape (N52°03′44.0″ E16°49′09.0″), 40 km from the spore sampler. Observations were executed in accordance with standard regulations prescribed by the Polish Meteorological authorities. The meteorological elements taken into consideration for the assessment of the effects of weather conditions on airborne fungal spores were: daily precipitation (mm), wind velocity (m s^−1^), wind direction (°), relative humidity (%), average air temperature (°C), air pressure (hPa) and total radiation (W m^−2^). Statistical analyses done in this study were based on regression, correlation methods and coefficients.

## Results

### Analysis of climate changes

**Fig. 1:**
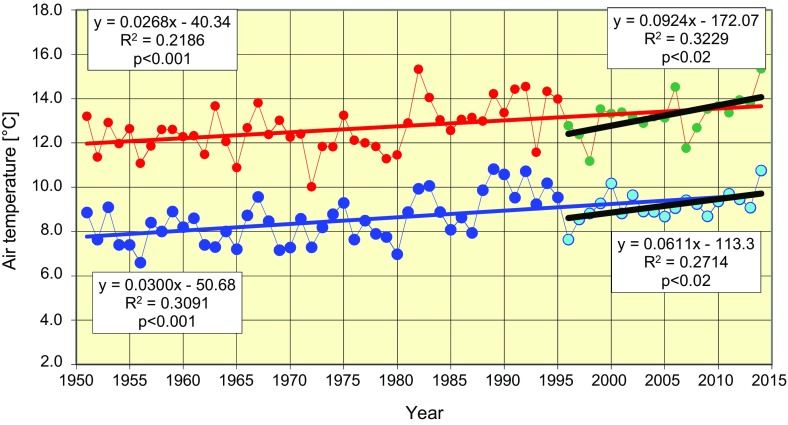
Trends in air temperature changes in the years 1951–2014 in Poznan; annual (*blue*) and average temperature for the period July–November (*red*). *Black lines* show the trends in the period 1998–2014. Correlation equations and determination coefficients on the *left-hand side* pertain to the years 1951–2014, and those on the *right-hand side* pertain to the period 1998–2014

There was a clear upward trend in the mean annual air temperature over the years 1950–2014 (Fig. 1). In this period temperature rose from 7.8 to 9.7 °C, an increase of 0.30 °C per decade was observed. In the period July to November, crucial for the development of pseudothecia (the fruiting bodies of *L. maculans*–*L. biglobosa*), the formation of ascospores and their release, temperature increased from 11.9 to 13.6 °C. Thus, the 0.26 °C per decade rate of temperature increase was slightly lower. This average is, however, a misleading value because mean annual temperature as well as mean air temperature between July and November did not change until 1980. An increase in air temperature has been observed since then, and increase over the period (1998–2014) covered by the current study amounted to an annual increase of 0.6 or 0.9 °C over the July–November season (right-hand side of Fig. 1).

**Fig. 2:**
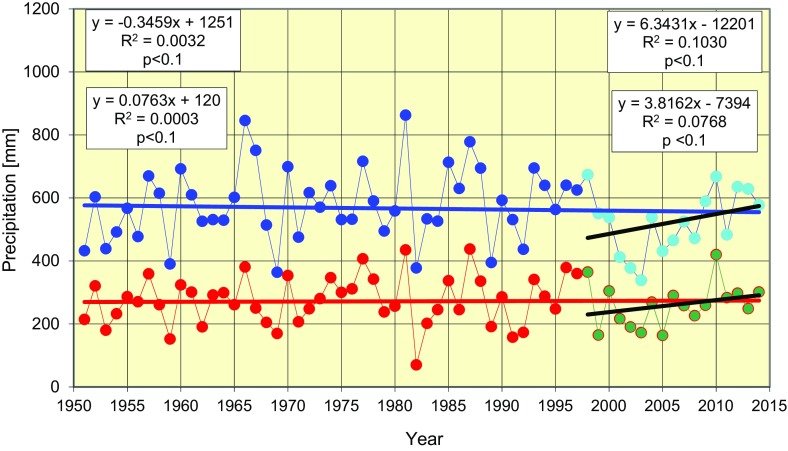
Trends in precipitation changes during the years 1951–2014 in Poznan; annual (*blue*) and average values for the season July–November (*red*). *Black lines* show these trends over the period 1998–2014. Correlation equations and determination coefficients on the *left-hand side* are for the years 1951–2014, and these on the *right-hand side* are for the period 1998–2014

No change was observed in average yearly precipitation in the years 1950–2014 (Fig. 2). However, during the period (1998–2014; right-hand side of Fig. 2) under study, a marked increase in annual rainfall, particularly during the months July–November, was found. This increase in yearly precipitation amounted to 38 mm per decade, and in the period July–November, it was 60 % higher and reached 63 mm per decade.

### Effect of climate changes on sexual sporulation of *L. maculans*–*L. biglobosa* species complex

Changes in the life cycle of *L. maculans*–*L. biglobosa* species complex during the 17 years of the current study (1998–2014) involved the following parameters: (1) the date of the first observation of ascospores in air samples, (2) the number of days with ascospores present in collected air samples, (3) the date of the maximum concentration of the ascospores m^−3^ of the air and (4) the cumulative number of the ascospores per season.

**Fig. 3:**
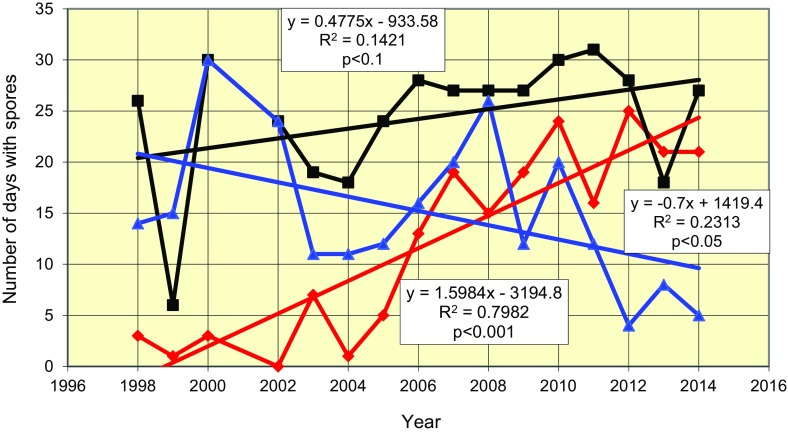
Variations in the number of days with ascospores of *L. maculans*–*L. biglobosa* species complex over the period 1998–2014 during September (*red*), October (*violet*) and November (*blue*)

In consecutive years, we have observed an increasing number of days in September and October with the occurrence of the ascospores of *L. maculans*–*L. biglobosa* species complex in the air (Table 1; Fig. 3). This trend was the fastest in September, when the average rate amounted to 1.6 days per year. In October, the number of days with ascospores captured from air samples increased by 0.4 days per year. At the same time, the number of days with spores in November decreased at a rate of 0.8 days per year.

**Table 1:** Summary of aerobiological data obtained for ascospores of the *L. maculans*–*L. biglobosa* species complex in air samples from Poznan, Poland, over the 17 years (1998–2014) of study

Period	Parameter	Year															
		1998	1999	2000	2002	2003	2004	2005	2006	2007	2008	2009	2010	2011	2012	2013	2014
Autumn season (1 Sept–30 Nov)	Number of days with ascospore detection in the air	43	21	63	48	37	30	41	57	66	68	58	74	59	57	47	53
	Date of the first ascospore detection	28 Sept	25 Sept	25 Sept	1 Oct	10 Sept	23 Sept	22 Sept	18 Sept	8 Sept	14 Sept	9 Sept	7 Sept	15 Sept	6 Sept	8 Sept	8 Sept
	Date of the detection of maximum ascospore concentration	13 Nov	14 Nov	29 Oct	18 Nov	1 Oct	20 Oct	27 Oct	27 Oct	11 Oct	17 Oct	30 Sept	25 Sept	30 Sept	21 Sept	1 Oct	23 Sept
	The highest daily mean concentration of ascospores m^−3^ of air	7.71	3.89	34,.20	42.78	9.17	9.86	4.03	43.05	105.83	48.61	43.75	43.75	86.38	67.63	64.86	43.75
	Sum of daily mean ascospore concentrations m^−3^ of air	106.90	19.10	247.00	605.00	70.40	46.20	34.10	367.90	1260.60	251.50	385.00	510.50	1049.70	659.00	287.10	308.90
	Mean ascospore concentrations m^−3^ of air	1.18	0.21	2.71	6.65	0.77	0.51	0.37	4.04	13.85	2.76	4.23	5.61	11.53	7.24	3.15	3.39
1–30 September	Number of days with ascospore detection in the air	3	1	3	0	7	1	5	13	19	15	19	24	16	25	21	21
	The highest daily mean concentration of ascospores m^−3^ of air	2.15	0.07	3.89	0	5.56	0.14	0.69	38.47	77.22	26.8	43.75	43.75	86.38	67.63	10.83	43.75
	Sum of daily mean ascospore concentrations m^−3^ of air	4.20	0.10	4.60	0	13.10	0.10	1.90	115.80	317.30	64.30	113.70	256.10	446.60	509.70	50.60	187.80
	Mean ascospore concentrations m^−3^ of air	0.14	0	0.15	0	0.44	0	0.06	3.86	10.58	2.14	3.79	8.54	14.89	16.99	1.69	6.26
1–31 October	Number of days with ascospore detection in the air	26	6	30	24	19	18	24	28	27	27	27	30	31	28	18	27
	The highest daily mean concentration of ascospores in m^−3^ of air	6.11	1.39	34.72	39.23	9.17	9.86	4.03	43.05	105.83	48.61	29.16	30.69	83.61	30.83	64.86	43.75
	Sum of daily mean ascospore concentrations m^−3^ of air	65.80	3.60	162.90	326.8	46.70	25.40	25.80	206.50	691.30	164.60	205.80	135.80	586.10	1450	230.50	116.50
	Mean ascospore concentrations m^−3^ of air	2.12	0.12	5.26	10.54	1.51	0.82	0.83	6.66	22.3	5.31	6.64	4.38	18.91	4.68	7.44	3.76
1–30 November	Number of days with ascospore detection in the air	14	15	30	24	11	11	12	16	20	26	12	20	12	4	8	5
	The highest daily mean concentration of ascospores m^−3^ of air	7.71	3.89	14.03	42.78	3.33	8.19	1.25	6.25	61.25	2.08	40.14	21.39	7.78	3.19	2.08	1.67
	Sum of daily mean ascospore concentrations m^−3^ of air	37.00	15.50	79.50	278.20	10.70	20.70	6.40	45.60	251.90	22.60	65.40	118.60	16.90	4.30	60	4.60
	Mean ascospore concentrations m^−3^ of air	1.23	0.52	2.65	9.27	0.36	0.69	0.21	1.52	8.40	0.75	2.18	3.95	0.56	0.14	0.20	0.15

**Fig. 4:**
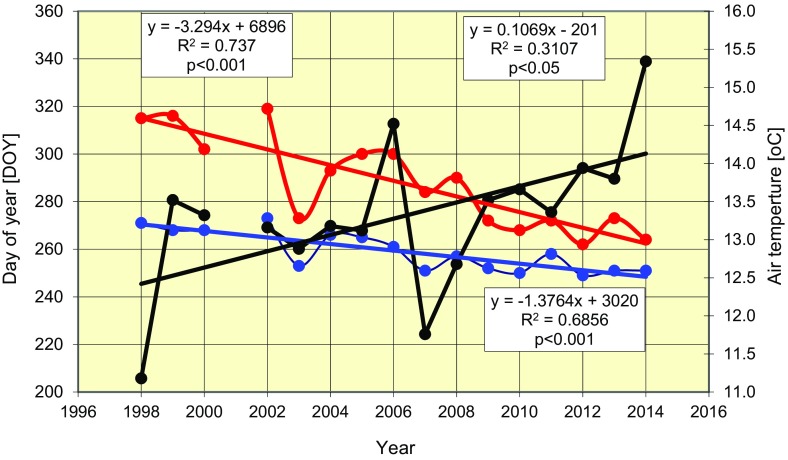
Acceleration of the dates (month) of the first detection of the ascospores of *L. maculans*–*L. biglobosa* species complex and of maximal ascospore concentrations in air (ind  m^3^) samples. *Violet* changes of air temperature, *blue* changes of date of the first detection of ascospores, *red* changes of date of maximal ascospore concentrations

**Fig. 5:**
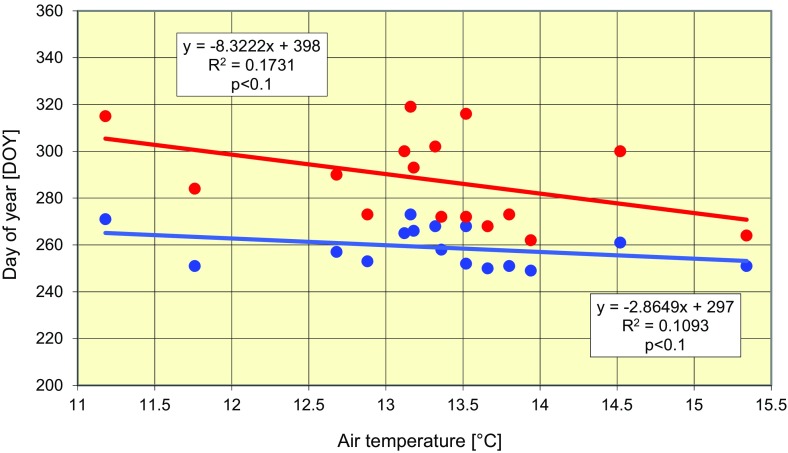
Interdependence between the dates of the first detection of the ascospores of *L. maculans*–*L. biglobosa* species complex (*blue*) or the date with the maximum ascospore concentration in the air (ind m^3^) (*red*) and air temperature

The increase in air temperature coincided with the date of the first observation of fungal spores in air samples, as well as the date of mass ascospore release; both dates occurred earlier as temperature increased (Fig. 4). The date of the first observation of *L. maculans*–*L. biglobosa* species complex ascospores changed from the 270th to 248th day of the year (DOY) (i.e. by 22 days), translating into an average rate of 1.37 days per year. Moreover, the date of mass release of ascospores shifted by 50 days from 315th to the 265th DOY at an average of 3.3 days per year. During this period, the average air temperature increased from 12.3 to 14.3 °C (1.7 °C) for July to November at the rate of 0.1 °C per year. Thus, a 1 °C change in mean air temperature during the period under study resulted in accelerated shortening of the date of first ascospore release by about 3 days and of the date of their mass concentration in air samples by about 8 days (Fig. 5).

**Fig. 6:**
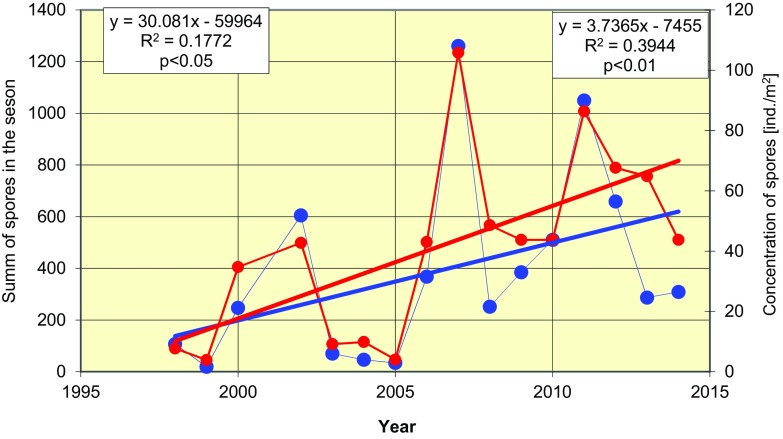
Variations in the seasonal abundance of the ascospores of *L. maculans*–*L. biglobosa* species complex (*blue*) and maximal monthly concentration of spores m^−3^ of the air (*red*), in the period 1998–2014. Correlation equation and determination coefficient on the *left-hand side* relate to seasonal sum, and those on the *right-hand side* refer to the concentration of spores in air (ind m^3^) samples

**Fig. 7:**
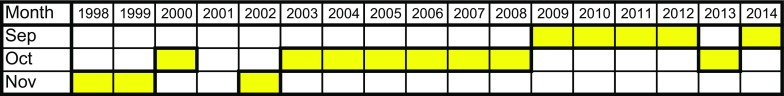
Months with the maximum concentrations of the ascospores of *L. maculans*–*L. biglobosa* species complex in air samples during each year (1998–2014) of the current study (*yellow*)

Data analyses further showed that besides earlier dates for the first and maximal ascospore releases, cumulative number of released ascospores, as well as the concentrations of these fungal spores, also increased in air samples (Fig. 6). Thus, the maximum release of spores in 1998–2002 was observed in November (except 2000), in October in 2003–2008 and in September for most of 2009–2014, with the exception of 2013 when it was October (Fig. 7).

## Discussion

The analyses done in the current study on the dataset collected in central-west Poland over 17 years (1998–2014), as a part of the SPEC system (Jedryczka et al. 2008), demonstrated a strong dependence of the development of the perfect stage of *L. maculans*–*L. biglobosa* species complex on average air temperature and precipitation. The influence of these parameters was observed both in average yearly effects and also the monthly effects over a period that was crucial for the pathogens’ life cycle (July–November). In this period a very large acceleration of fungal developmental processes was related to air temperature. This weather parameter has increased since the 1980 s and was observed to be on the upward trend over the period of the current study (1998–2014). Temperature increase is well known to have a significant impact on the behaviour and biology of living organisms and on physiological and biochemical processes taking place in the biosphere (Drake 2000; Stevens 2001). In the special case of the fungal pathogen species complex currently under study, a temperature increase of nearly 2 °C over 17 years effected alterations in the rates of the formation and maturation of fruiting bodies of *L. maculans* and *L. biglobosa* and the subsequent release of the ascospores. The process of more intensive and accelerated sporulation was concomitant with the rising pattern of rainfall increase over this period (1998–2004). Changes of hygrothermal conditions towards increasingly warm and moist weather were favourable for growth and development of *L. maculans*–*L. biglobosa* species complex.

Very significant increases in the number of days with airborne ascospores in September and a marked decline in November were indicative of a displacement of maximal sporulation intensity from late to early autumn. This could have resulted in greatly increased potential of these pathogens to infect plants, occasioned by increased host vulnerability to the disease as well as by the longer time for disease development in warmer autumn days. The rate of shortening of the days to maximum ascospore release was higher than rate of shortening of the days to the first ascospore release, thereby resulting in a much shorter time lapse between these two dates. The difference between the date of maximum and the first date of sporulation of the teleomorphs of members of the *L. maculans*–*L. biglobosa* species complex was 45 days in 1998, but only 17 days in 2014. This, however, may also result from the monitoring time starting always not earlier than 1 September, due to sowing/germination of oilseed rape in Wielkopolska region.

It is reasonable to infer, judging by the data obtained in the current study, that air temperature was the most significant determinant of this phenomenon. We have found that average air temperature and precipitation for the months of July to November, the time for pseudothecial maturation and ascospore release in *L. maculans* and *L. biglobosa*, increased during the years under study at the rates of 0.1 °C and 6.3 mm per year, respectively. Concomitantly, the date of the year for the first detection of ascospores shifted from 270 to 248 DOY, which translates into 22 days of acceleration. The process of the maximum release of ascospores was altered by nearly 2.5 times, from 315 to 265 DOY (50 days of the year).

The results obtained in this study are in agreement with the forecast for the UK elaborated by Evans et al. (2008), using multi-site data that were collected over a 15-year period. The long-term field evaluations combined with climate change scenarios enabled these workers to develop and validate a weather-based model for forecasting the severity of phoma stem canker disease on oilseed rape across the UK. The authors predicted that epidemics of this disease will not only increase in severity in England but also move northwards by the 2020 s. The predicted increase in stem canker epidemics on oilseed rape in southern England and a spread of *L. maculans* into Scotland were based on increased temperatures associated with warmer winters and dryer summers. The urgent need to predict the effects of climate change on other plant diseases was suggested and the authors stressed that the above-mentioned changes take place in delicately balanced agricultural ecosystems (Evans et al. 2008).

A very important consideration for arable agriculture were the potentially hazardous systematic and cumulative increase in ascospore abundance during the cropping season as well as increases in their concentration in air samples. Some of the data obtained during the current study indicate the need for further research to resolve some currently intractable temporal associations. For example, results obtained during this study in 2007 and 2011 show the possibility of crucial roles that can be attributed to daily or perhaps hourly, temperatures or single rain events. The year 2007 was cool with average precipitation, while the year 2011 had average temperature variations but was dry. However, in both years, two of the highest ascospore counts and the two highest ascospore concentrations were observed. It is, therefore, possible that in certain cases, extremes of meteorological elements had greater importance than their averaged monthly values. It would be also of great interest to study the effect of climate change separately for each of the two *Leptosphaeria* species responsible for blackleg of oilseed rape. Such studies are now technically possible, due to molecular biological tools that permit the discrimination of DNA present in ascospores, mycelial fragments and pycnidiospores of *L. maculans* and *L. biglobosa* (Kaczmarek et al. 2008, 2012; Piliponyte-Dzikiene et al. 2014). Such possibility is available even in respect to avirulence variants of *L. maculans* (Van de Wouw et al. 2010; Kaczmarek et al. 2014).

The relationships between fungal spores of *Cladosporium* and *Alternaria* in aeroplankton and selected meteorological factors have been extensively described by Grinn-Gofroń (2009a, b). Studies on ascospore release by *Leptosphaeria* spp. that were carried out using spore samplers located on roof tops demonstrated that ascospore numbers increased on days with rainfall (Burge 1986). In Crete, the ascospores of the genus *Leptosphaeria* were the most numerous among all spores of the ascomycetes that were detected and constituted nearly 7 % of the air mycoflora and nearly half of all ascospores present in the air. It was suggested that such abundance of *Leptosphaeria* ascospores was connected with the microclimate of this island (Gonianakis et al. 2006).

In case of studies concerning *L. maculans* and *L. biglobosa*, Toscano-Underwood et al. (2003) demonstrated that the maturation of pseudothecia on oilseed rape stem debris was influenced by both air temperature and humidity. Earlier experiments that were done at four locations in Poland over 4 years (2005–2008) showed that higher moisture content of senescing, but still living, stems play a crucial role in the early commencement of the ascospore maturation season and the maximum release of ascospores (Dawidziuk et al. 2012b). Furthermore, these authors also found that similar patterns of pseudothecial development occurred in both fungal species of *Leptosphaeria*, causing stem canker symptoms. Monitoring of ascospores in air samples, combined with molecular biological detection of *L. maculans* and *L. biglobosa* using quantitative PCR methods, showed no distinct differences between these two pathogens in relation to patterns and earliness of spore release (Kaczmarek and Jedryczka 2011).

We have demonstrated that the conduciveness of climate for ascospore release by *L. maculans* and *L. biglobosa* increased with increases in temperature and precipitation. The implications of these interactions between pathogen sporulation and the physical environment bring evidence for the strong impact of the current changes of weather on the increase in stem canker severity in oilseed rape in Poland. These observations complement the already-overwhelming scientific empirical evidence for an increase in the frequency of extreme weather events. They underpin the need for more basic and applied research to further explore the effects of climate change on plant pathogen populations, particularly the impact on the biology of pathogenic fungi with wind-dispersed spores in order to prepare ways (by both adaptation and mitigation) to prevent and alleviate their adverse effects on agricultural production.
